# Correction: Phylogenomic reclassification of *Cellulophaga* species to *Paracellulophaga* gen. nov. and description of *Allocellulophaga tsushimaensis* gen. nov., sp. nov., a novel bacterium from coastal seawater of Tsushima Island, Japan

**DOI:** 10.1007/s10482-026-02312-y

**Published:** 2026-05-05

**Authors:** Yu Nakajima, Shu-Kuan Wong, Yuki Muramatsu, Marie Johanna Cuadra, Kei Zenimoto, Keigi Ou, Hiroshi Xavier Chiura, Keiji Nakamura, Yasuhiro Gotoh, Tetsuya Hayashi, Yasuyoshi Nakagawa, Susumu Yoshizawa

**Affiliations:** 1 Japan Agency for Marine-Earth Science and Technology (JAMSTEC), Research Institute for Marine Resource Utilization (MRU), 2-15 Natsushima-Cho, Yokosuka, Kanagawa Japan; 2 Arctic Environment Research Center, National Institute of Polar Research, 10-3 Midoricho, Tachikawa, Tokyo Japan; 3 Biological Resource Center, National Institute of Technology and Evaluation (NBRC), 2-5-8 Kazusakamatari, Kisarazu, Japan; 4 Department of Biological Sciences, College of Science and Mathematics, Mindanao State University-Iligan Institute of Technology, Andres Bonifacio Avenue, Tibanga, 9200 Iligan City, Philippines; 5 LLC FlatHour, Kamiagatamachi Shitaru 3021, Tsushima, Nagasaki Japan; 6 Graduate School of Frontier Sciences, The University of Tokyo, 5-1-5 Kashiwanoha, Kashiwa, Chiba, Japan; 7 Atmosphere and Ocean Research Institute, The University of Tokyo, 5-1-5 Kashiwanoha, Kashiwa, Chiba Japan; 8 Department of Bacteriology, Faculty of Medical Sciences, Kyushu University, Fukuoka-Shi, Fukuoka, Japan

**Correction to: Antonie van Leeuwenhoek (2026) 119(4), 66** 10.1007/s10482-026-02275-0.

In the original publication of the article, the protologue presented in the “Emended description of *Paracellulophaga*” section was insufficient. By including culture collection numbers, we clarified that the type species is available from two different countries, and we also added descriptions for three additional species that should be included in the genus (description of comb. nov.). Furthermore, regarding motility in the description of *Paracellulophaga* gen. nov., we corrected the statement to indicate that the genus exhibits gliding motility rather than being non-motile.

The original article is published as below:

**Emended description of Paracellulophaga Johansen (Johansen et al. 1999)**

*Paracellulophaga* (Pa.ra.cel.lu.lo’pha.ga. Gr. pref. *para*, beside, alongside; N.L. neut. n. cellulosum, cellulose; Gr. inf. v. phageîn, to eat; N.L. fem. n. *Paracellulophaga*, cellulose eater alongside, referring to the endocellulase enzyme activity and close phylogenetic relationship to the genus *Cellulophaga*.)

Description of the genus *Paracellulophaga* is partially based on that given by (Johansen et al. 1999; Bowman 2000; Nedashkovskaya et al. 2004; Kahng et al. 2009).

Cells are rod-shaped, Gram-staining-negative, catalase positive, and non-motile. Growth occurs at pH 7–8 and 2–30 °C under aerobic condition. The predominant respiratory quinone is MK-6. The major fatty acids are C_15:0_, iso-C_15:0_, iso-C_15:0_ 3-OH, iso- C_15:1_ *ω10c*, iso-C_16:0_ 3-OH, C_16:1_ *ω7c*, iso-C_17:0_ 3-OH, and iso-C_17:1_ *ω7c*. The DNA G + C content is 33.0%. The type species is *Paracellulophaga baltica.*

The correct protologue for this novel taxon should read:

**Description of** ***Paracellulophaga*** **gen. nov.**

*Paracellulophaga* (Pa.ra.cel.lu.lo’pha.ga. Gr. prep. *para*, beside, alongside; N.L. neut. n. *Cellulophaga*, a bacterial genus name; N.L. fem. n. *Paracellulophaga*, an organism phylogenetically related to the genus *Cellulophaga*.)

Cells are rod-shaped, Gram-stain-negative, catalase-positive and motile by gliding. Growth occurs at pH 5.5–10 and 2–35 °C under aerobic conditions. The predominant respiratory quinone is MK-6. The major fatty acids are C_15:0_, iso-C_15:0_, iso-C_15:0_ 3-OH, iso-C_15:1_ *ω10c*, iso-C_16:0_ 3-OH, C_16:1_ *ω7c*, iso-C_17:0_ 3-OH and iso-C_17:1_ *ω7c*. The genus is a member of the family *Flavobacteriaceae*. The type species is *Paracellulophaga baltica*.

**Description of** ***Paracellulophaga baltica*** **comb. nov.**

*Paracellulophaga baltica* (bal'ti.ca. M.L. fem. adj. *baltica*, pertaining to the Baltic Sea.)

Basonym: *Cellulophaga baltica* Johansen et al. 1999.

Characteristics of the species are as described by Johansen et al. (1999).

The type strain NN015840^T^ (= ATCC 700862^T^ = DSM 24729^T^) was isolated from the surface of the brown alga *Fucus serratus* collected from the Baltic Sea. The G + C content of this species is 33.0 mol%.

**Description of** ***Paracellulophaga algicola*** **comb. nov.**

*Paracellulophaga algicola* (al.gi’co.la. L. fem. n. *alga*, *alga*; L. masc./fem. n. incola, inhabitant, dweller; N.L. masc./fem. n. *algicola*, algae-dweller).

Basonym: *Cellulophaga algicola* Bowman 2000.

Characteristics of the species are as described by Bowman (2000).

The type strain IC166^T^ (= ACAM 630^T^ = DSM 14237^T^) was isolated from the surfaces of the chain-forming sea-ice diatom. The G + C content of this species is 36 to 38 mol%.

**Description of** ***Paracellulophaga tyrosinoxydans*** **comb. nov.**

*Paracellulophaga tyrosinoxydans* (ty.ro.sin.o’xy.dans. N.L. fem. n. *tyrosina*, tyrosine; N.L. pres. part. *oxydans*, oxidizing; N.L. fem. part. adj. *tyrosinoxydans*, pertaining to the ability to oxidize tyrosine).

Basonym: *Cellulophaga tyrosinoxydans* Kahng et al. 2009

Characteristics of the species are as described by Kahng et al. (2009).

The type strain EM41^T^ (= KCTC 22297^T^ = DSM 21164^T^) was isolated from seawater on the eastern coast. The G + C content of this species is 33.5 mol%.

**Description of** ***Paracellulophaga pacifica*** **comb. nov.**

*Paracellulophaga pacifica* (pa.ci’fi.ca. L. fem. adj. *pacifica*, peaceful, referring to the Pacific Ocean, from which the organism was isolated).

Basonym: *Cellulophaga pacifica* Nedashkovskaya et al. (2004)

Characteristics of the species are as described by Nedashkovskaya et al. (2004).

The type strain KMM 3664^T^ (= JCM 11735T = LMG 21938^T^) was isolated from seawater in the Gulf of Peter the Great, Sea of Japan. The G + C content of this species is 32 to 34 mol%.
